# The detection of anti-dengue virus IgM in urine in participants enrolled in an acute febrile illness study in Puerto Rico

**DOI:** 10.1371/journal.pntd.0007971

**Published:** 2020-01-29

**Authors:** Elba Caraballo, B. Katherine Poole-Smith, Kay M. Tomashek, Brenda Torres-Velasquez, Luisa I. Alvarado, Olga D. Lorenzi, Carmen Ramos, Jessica Carrión, Elizabeth Hunsperger

**Affiliations:** 1 University of Puerto Rico, UPR- Comprehensive Cancer Center(UPRCCC), Division of Cancer Biology, San Juan, Puerto Rico; 2 Armed Forces Research Institute of Medical Sciences (AFRIMS), Bangkok, Thailand; 3 National Institutes of Health (NIH) National Institute of Allergy and Infectious Diseases (NIAID), Division of Microbiology and Infectious Diseases (DMID), Office of Clinical Research Resources (OCRR); 4 Centers for Disease Control and Prevention (CDC), Division of Vector Borne Diseases, San Juan, Puerto Rico; 5 Ponce Health Science University, Ponce, Puerto Rico; 6 CDC, Division of Global Health Protection, Epidemiology, Surveillance, Informatics, and Laboratory Branch, CDC-Kenya; University of Pittsburgh, UNITED STATES

## Abstract

**Background:**

Dengue is an important arboviral disease with about 100 million dengue cases per year, of which, ~5% result in severe disease. Clinical differentiation of dengue from other acute febrile illnesses (AFI) is difficult, and diagnostic blood tests are costly. We evaluated the utility of anti-DENV IgM in urine to identify dengue cases among AFI patients enrolled in a clinical study.

**Methods:**

Between May 2012-March 2013, 1538 study participants with fever for ≤7 days were enrolled, a medical history was obtained, and serum and urine specimens were collected. Serum was tested for DENV RNA and anti-DENV IgM. Urine was tested for anti-DENV IgM, and its sensitivity and specificity to detect sera laboratory-positive dengue cases were calculated. We evaluated if urine anti-DENV IgM positivity early (≤5 days post-illness onset [DPO]) and late (6–14 DPO) in the clinical course was associated with dengue severity.

**Results:**

Urine anti-DENV IgM sensitivity and specificity were 47.4% and 98.5%, respectively, when compared with serum anti-DENV IgM ELISA results, and 29.7% and 91.1% when compared with serum rRT-PCR results. There was no correlation between urine anti-DENV IgM positivity and patient sex or pre-existing chronic disease. Early in the clinical course, a significantly higher proportion of those who developed dengue with warning signs had anti-DENV IgM in their urine when compared to those without warning signs (20.4% vs. 4.3%). There was no difference in the proportion with urine anti-DENV IgM positivity between severity groups late in the clinical course.

**Conclusion:**

While detection of urine anti-DENV IgM lacked adequate diagnostic sensitivity, it is a highly specific marker for laboratory-positive dengue, and its presence early in the clinical course may distinguish those with more severe disease. Further assessment of urine anti-DENV IgM by DPO is warranted to determine its utility as an early diagnostic (and possibly prognostic) marker for dengue.

## Author summary

Dengue viruses (DENV) are transmitted to humans by infected mosquitoes and the disease, known as dengue, occurs throughout the tropics and subtropics. Dengue diagnostic tests rely on a blood sample taken within two weeks of illness onset. Our primary objective was to determine if urine (an easy sample to collect) could be used to detect dengue cases among patients with an acute febrile illness (AFI). Our second objective was to determine if severe dengue was associated with the detection of anti-DENV IgM antibodies in the urine. We found that detecting urine anti-DENV IgM in AFI patients meant that they likely had dengue, that is, it was a highly specific diagnostic test (few false positives). However, a negative urine test did not rule out dengue, that is, it was not a very sensitive test. Anti-DENV IgM in urine was more likely to be detected in the first five days of illness among patients who later developed severe dengue than those with less severe dengue. These findings suggest that the presence of anti-DENV IgM in urine may be useful to diagnose dengue and may indicate more severe disease if done early in the illness. More studies are needed to confirm these results.

## Introduction

Dengue is a disease that occurs in pandemic proportions throughout tropical and subtropical regions of the world and affects persons living in urban and rural areas. It has been estimated that there are 300 million dengue virus (DENV) infections per year and 100 million dengue cases [1]. Dengue initially presents as an acute febrile illness (AFI) that can be difficult to diagnose clinically and differentiate from other AFIs such as malaria, leptospirosis, influenza, and chikungunya; however, even if dengue is suspected and diagnosed early, a patient’s clinical course and outcome is unpredictable. After a two to seven day AFI, most patients with dengue will improve; however, about 5% of dengue cases will develop more severe disease characterized by a plasma leakage syndrome with effusions, acute respiratory distress, and hypovolemic shock; severe bleeding; and/or organ impairment [2, 3]. To ensure timely and appropriate clinical management of dengue cases, a simple, rapid laboratory diagnostic assay is needed.

Currently most diagnostic laboratory tests for dengue require serum specimens to detect DENV nucleic acid during the acute phase of the illness (i.e., days post-illness onset [DPO] ≤5) or anti-DENV IgM during the convalescent phase (DPO 6–14). A test that uses a non-invasive specimen such as urine could be of benefit in cases where serum is difficult to obtain (*e*.*g*., young children and elderly), and in fact, urine has been previously studied as a surrogate specimen for serum [4, 5]. In addition, urine is relatively easy and inexpensive to collect, and it is often collected in patients presenting with an AFI [6]. Previous studies where urine was evaluated as a diagnostic specimen for dengue have provided varied results dependent on the immunoglobulin tested (*i*.*e*., IgA, IgM, IgG) [4, 5, 7, 8]. This variance could be due to differences in immunoassay sensitivity, timing of specimen collection during the clinical course, and/or the age of the patient population studied. By using a highly sensitive anti-DENV IgM diagnostic immunoassay, and collecting urine during the first five days of illness (acute) and 6–14 days after illness onset (convalescent), we sought to investigate the utility of urine as a diagnostic specimen for dengue among patients of all ages [9].

In most healthy individuals, IgM is not detected in the urine. However, in diseases in which there is transient acute or chronic kidney injury resulting in a change in glomerular capillary wall permeability, large proteins, including IgM, may be found in the urine [10–12]. In the case of dengue, acute kidney injury (AKI) and acute renal failure are potential medical complications associated with prolonged shock and/or rhabdomyolysis [13–17]. A study of IgA nephropathy [18] and a few case reports describing hematuria, proteinuria and acute glomerulonephritis among dengue patients suggest that anti-DENV IgM may be detected in the urine of some dengue patients [19, 20]. In fact, proteinuria has been found in a significant proportion (up to about 30%) of dengue patients, and a few recent studies have evaluated proteinuria as a predictor of severe dengue [21–24].

In our study, the main objective was to determine whether urine can be used as a surrogate diagnostic specimen for the detection of laboratory-positive dengue cases among AFI patients. We did this by determining the sensitivity and specificity of a urine anti-DENV IgM enzyme-linked immunosorbent assay (ELISA) when compared with a serum anti-DENV IgM ELISA and a serotype-specific real-time, reverse transcription-polymerase chain reaction assay (rRT-PCR). Our second study objective was to determine if the presence of urine anti-DENV IgM was a predictor of severe dengue among laboratory-positive dengue cases.

## Methods

### Study population

We used specimens collected from 1538 study participants enrolled between May 2012-March 2013, of an AFI study conducted in Ponce, Puerto Rico [25]. As previously described, patients of all ages were asked to participate if they had an AFI defined by presence of a fever or history of fever lasting ≤7 days. Participants who were discharged to home after enrollment were asked to return 7 to 10 DPO. During this follow-up visit, the study nurse completed another form to document any healthcare services received and signs and symptoms that developed after enrollment. Participants admitted to the hospital at enrollment had their hospital course, including treatment received, results of clinical laboratory and radiologic investigations, and disease manifestations, recorded on a hospital admission summary form. Study participants had blood and urine specimens collected at enrollment and follow-up visits, or before hospital discharge. Blood and urine specimens were kept at 4°C until transported to the Centers for Disease Control and Prevention (CDC), Dengue Branch in San Juan, Puerto Rico for testing.

### Ethics statement

Prior to enrollment, informed consent was administered in accordance with Puerto Rico law (Article 13, Section 13, Regulation 7617 of the Office of Patient Ombudsman, Act #194). We obtained a written informed consent from eligible adults >20 years old and emancipated minors 14–20 years old. For non-emancipated minors (14–20 years old) a written informed assent was obtained and written informed consent was obtained from the parents or guardians. For children 7–13 years old verbal informed assent was obtained and written informed consent was obtained from the parents or guardian, and the parents or guardian of children <7 years old. After informed consent was obtained, study nurses completed a form to document vital signs, symptoms, history of chronic disease, and results of routine clinical laboratory tests. The study protocol was reviewed and approved by the Institutional Review Boards at the CDC and the Ponce School of Medicine (SEDSS protocol no. 6214).

### Laboratory diagnostic testing

Serum specimens collected ≤6 DPO were tested by rRT-PCR to detect DENV nucleic acid as previously described [26]. Serum specimens collected ≥4 DPO were tested for the presence of anti-DENV IgM by DENV Detect IgM ELISA (InBios International, Inc., Seattle, WA) per manufacturer’s instructions. For the purpose of this analysis, we defined acute phase specimens as ≤5 DPO and convalescent phase specimens were defined as 6–14 DPO. Prior to testing urine, the positive control from the ELISA kit was spiked into urine and we observed acceptable recovery. We proceeded to test paired undiluted urine specimens from the acute and convalescent phase for anti-DENV IgM using DENV Detect IgM ELISA (InBios) per the manufacturer’s instructions. Urine anti-DENV-IgM results were analyzed by DPO and disease severity as defined using the 2009 World Health Organization (WHO) guidelines [27].

### Definitions

**Laboratory-positive dengue case:** a study participant who met one or more of the following criteria: 1) DENV nucleic acid detected in a serum specimen by rRT-PCR; 2) anti-DENV IgM antibodies present in serum with an Immune Status Ratio ≥2.84 as defined by the manufacturer.

**Laboratory-negative dengue case:** a participant with no DENV nucleic acid detected in a serum specimen by rRT-PCR collected ≤6 DPO and no anti-DENV IgM detected in a serum specimen collected ≥4 DPO.

**Case of dengue:** an AFI with two or more of the following signs and symptoms: nausea, vomiting, rash, aches and pains, tourniquet test positive, or leucopenia as defined by the 2009 WHO guidelines [27]. Dengue with warning sign cases met the case definition for dengue plus had one or more warning signs for severe dengue including: severe abdominal pain, persistent vomiting, mucosal bleed, lethargy or restlessness, liver enlargement ≥2 cm, and rising hematocrit with rapid decrease in platelet count.

**Case of severe dengue:** an AFI with one or more of the following as defined by 2009 WHO guidelines [27]: 1) significant plasma leakage leading to shock or fluid accumulation resulting in acute respiratory distress; 2) severe bleeding with hemodynamic instability that requires fluid replacement and/or blood transfusion or any bleed into a vital organ such as an intracranial bleed; or 3) severe organ impairment such as acute liver failure, myocarditis, or neurologic disease.

### Statistical analysis

Urine anti-DENV IgM ELISA results were compared with serum anti-DENV IgM ELISA and rRT-PCR results to estimate with 95% CI the sensitivity and specificity of the urine assay. Patient characteristics, including age, sex, and pre-existing chronic conditions that may be associated with a positive urine anti-DENV IgM result were evaluated using the Mann-Whitney-Wilcoxon and Wilson Score Interval to compare differences in median and proportions between groups, respectively. To evaluate if the presence of anti-DENV IgM in urine was an indicator of disease severity, Wilson Score Interval was used for the differences in proportions of participants with positive urine by clinical severity group (23).

## Results

Between May 2012 and March 2013, 1,538 study participants were enrolled and 580 had laboratory-positive dengue. Of the 580 laboratory-positive dengue cases, 534 had urine specimens; 342 (64.0%) urine specimens were negative for anti-DENV IgM and 192 (36.0%) were positive (Table 1). Study participants with anti-DENV IgM positive urine were slightly older than those with anti-DENV negative urine (median 15 years vs. 13 years, *P* = 0.02). There was no significant difference in the proportion with anti-DENV IgM positive urine by sex. Similarly, when we compared the proportion of male and female participants with anti-DENV IgM positive urine by age group (*i*.*e*., pre-menstrual, reproductive age, and post-menopausal age groups), there were no significant differences (S1 Table).

**Table 1:** Participants’ demographics and clinical features. Comparison of demographic and clinical features by urine anti-DENV IgM status among laboratory-positive dengue cases.

	Anti-DENV IgM Positive N = 192		Anti-DENV IgM Negative N = 342		Diff, CI*
**Demographics**					
Age, median (range)	13	(0.6–68.0)	15	(0.0–88.0)	0.0185^£^
** **	**N**	**%**	**N**	**%**	** **
Age group, years	** **	** **	** **	** **	** **
<9	47	24.5	70	20.5	(0.04, -0.03 − 0.12)
9–44	129	67.2	237	69.3	(-0.02, -0.1 − 0.06)
45+	16	8.3	35	10.2	(-0.02, -0.07 − 0.04)
Female	92	47.9	150	43.9	(0.04, -0.05 − 0.13)
Chronic medical conditions					
Anemia	43	22.4	72	21.1	(0.01, -0.06 − 0.09)
Diabetes	8	4.2	23	6.7	(-0.03, -0.06 − 0.02)
High blood pressure	12	6.2	18	5.3	(0.01, -0.03 − 0.06)
Renal disease	1	0.5	1	0.3	(0, -0.01 − 0.03)
Sickle cell disease	1	0.5	1	0.3	(0, -0.01 − 0.03)
Reported Clinical Features					
Blood in urine	3	1.6	8	2.3	(-0.01, -0.03 − 0.02)
Oranged colored urine	30	15.6	49	14.3	(0.01, -0.05 − 0.08)
**Clinical Diagnosis and Outcome**					
rRT-PCR positive dengue case	106	55.2	251	73.4	(-0.18, -0.27 − -0.1)
IgM serum positive dengue case	190	99.0	211	61.7	(0.37, 0.32 − 0.43)
Urinary tract infection/pyelonephritis	4	2.1	5	1.5	(0.01, -0.02 − 0.04)
Hospitalized	119	62.0	122	35.7	(0.26, 0.18 − 0.35)
**Blood Urea Nitrogen/Creatinine ratio**					
Intrarenal (<9.99)	40	20.8	57	16.7	(0.04, -0.03 − 0.11)
Normal or Post-renal (10–10.99)	99	51.6	142	41.5	(0.10, 0.01 − 0.19)
Pre-renal (>20)	28	14.6	28	8.2	(0.06, 0.01 − 0.13)
**Laboratory**					
Low serum albumin	78	40.6	79	23.1	(0.18, 0.09 − 0.26)
>5 red blood cells/HPF in urine	15	7.8	26	7.6	(0, -0.04 − 0.05)
Leukopenia	153	79.7	244	71.3	(0.08, 0.01 − 0.16)
Thrombocytopenia	102	53.1	103	30.1	(0.23, 0.14 − 0.31)
	**Median**	**Range**	**Median**	**Range**	
Platelet count(x 10^3)	95.0	(22.0–359.0)	130.0	(10.0–511.0)	<0.001^£^
White blood Cell (x 10^3)	2.8	(1.0–14.9)	3.5	(1.0–20.5)	<0.001^£^
Aspartate Transaminase	118.5	(21.0–999.0)	66.0	(10.0–1104.0)	<0.001^£^
Alanine Transaminase	82.0	(24.0–854.0)	58.0	(25.0–999.0)	<0.001^£^

*Note*: n = 534 true positive with an available urine sample

^£^ Mann Whitney Wilcoxon test to compare medians or *Wilson Score interval for difference of proportions with 95% confidence interval (CI)

There was no difference in the proportion of participants with anti-DENV IgM positive urine versus negative urine by reported pre-existing chronic medical condition including anemia, diabetes and high blood pressure (Table 1). There were only four participants with self-reported chronic renal disease or sickle cell disease, and therefore we were unable to evaluate urine anti-DENV IgM positivity for these conditions. In addition, there was no difference in urine positivity between those with and without self-reported clinical features including blood in urine or orange-colored urine. No association was found between having a urinary tract infection/pyelonephritis diagnosis and anti-DENV IgM positive urine, but only nine participants had this acute diagnosis.

Participants with anti-DENV IgM positive urine appeared to be more ill than those with negative urine (Table 1). For example, participants with anti-DENV IgM positive urine were more likely to be hospitalized than those with a negative urine (62.0% versus 35.7%, Wilson Score (WS) = 0.26, CI95 0.18–0.35). Similarly, a significantly higher proportion of participants with anti-DENV IgM positive than negative urine had clinical signs of severe disease including low serum albumin (40.6% vs. 23.1%, WS = 0.18, CI95 0.09–0.26), thrombocytopenia (53.1% vs. 30.1%, WS = 0.23, CI95 0.14–0.31), aspartate transaminase (median: 119 vs. 66, *P*<0.001) and alanine transaminase (median: 82 vs. 58, *P*<0.001). Last, a significantly higher proportion of participants with anti-DENV IgM positive urine than negative urine had a blood urea nitrogen/creatinine ratio categorized as pre-renal (14.6% vs. 8.2%, WS = .06, CI95 0.01–0.13), a finding that may be seen in severe dengue patients with plasma leakage and/or gastrointestinal bleeding.

### Sensitivity and specificity of urine anti-DENV IgM

Of the 1538 participants in the study, there were 1486 urine specimens available for the sensitivity and specificity analysis (Table 2). Of those, there were 106 laboratory-positive dengue cases with anti-DENV IgM detected in urine (i.e., true positives), and 1029 laboratory-negative dengue cases with no anti-DENV IgM detected in urine (i.e., true negatives). Almost all (99.0%) participants with anti-DENV IgM positive urine also had positive serum, whereas the opposite was true for rRT-PCR serum positivity.

**Table 2:** Diagnostic testing comparisons. Sensitivity and specificity of urine anti-DENV IgM by test type in serum.

Test comparison	True Positive (%)	True Negative (%)	False Positive (%)	False Negative (%)	Sensitivity (CI95)	Specificity (CI95)	Positive Predictive Value (CI95)	Negative Predictive Value (CI95)
DENV RT-PCR	106 (29.7)	1029 (91.1)	100 (8.9)	251 (70.3)	29.7 (25.0–34.7)	91.1 (89.3–92.7)	51.5 (44.4–58.5)	80.4 (78.1–82.5)
Anti-DENV IgM in Serum	190 (47.4)	1069 (98.5)	16 (1.5)	211 (52.6)	47.4 (42.4–52.4)	98.5 (97.6–99.2)	92.2 (87.7–95.5)	83.5 (81.4–85.5)
DENV RT-PCR or Anti-DENV IgM in Serum	192 (36)	938 (98.5)	14 (1.5)	342 (64)	36.0 (31.9–40.2)	98.5 (97.5–99.2)	93.2 (88.9–96.2)	73.3 (70.8–75.7)

*Note*: n = 1486 specimens that were true positive with an available urine sample.

The sensitivity and specificity of the urine anti-DENV IgM ELISA when serum rRT-PCR was used as reference method was 29.7 (CI95 25.0–34.7) and 91.1 (CI95 89.3–92.7), respectively. In comparison, there were 190 true positives and 1069 true negatives when a positive serum anti-DENV IgM ELISA was used as reference. The sensitivity and specificity when serum anti-DENV IgM was used as reference method was 47.4 (CI95 42.4–52.4) and 98.5 (CI95 97.6–99.2), respectively. When the comparator was a positive anti-DENV IgM ELISA and/or rRT-PCR in serum, there were 192 true positives and 938 true negatives, and the sensitivity and specificity of urine anti-DENV IgM was 36.0 (CI95 31.9–40.2) and 98.5 (CI95 97.5–99.2), respectively.

### Severity analysis

Of the 580 laboratory-positive dengue cases, 513 had both a urine specimen and met the WHO 2009 guidelines for disease classification (Table 3). Early in the clinical course (i.e., DPO <5), a statistically significant higher proportion of participants who had dengue with warning signs had anti-DENV-IgM detected in their urine than participants with dengue (20.4% vs. 4.3%, WS = -0.16, CI95–0.23 to -0.05). Conversely, late in the clinical course of infection over half of the participants with dengue (14/27, 51.9%), dengue with warning signs cases (115/189, 60.8%), and severe dengue (14/28, 50.0%) had anti-DENV IgM positive urine. When all cases are included in the analysis, dengue and dengue with warning signs remains statistically significantly different (*P* = .004).

**Table 3:** The presence of anti-DENV IgM in urine by dengue severity. Urine anti-DENV IgM results by dengue disease classification and day post-illness onset of specimen collection.

	Final Case Classification					
Day post-illness onset	Dengue	Dengue with warning signs	Severe dengue	(Diff, CI)*	(Diff, CI)Ɨ	(Diff, CI)ǂ
0–5	2/46 (4.3)	40/196 (20.4)	4/27 (14.8)	(-0.16, -0.23 **−** -0.05)	(-0.1, -0.29 − 0.03)	(0.06, -0.13 − 0.17)
>5	14/27 (51.9)	115/189 (60.8)	14/28 (50.0)	(-0.09, -0.28 − 0.10)	(0.02, -0.24 − 0.27)	(0.11, -0.08 − 0.30)
Total	16/73 (21.9)	155/385 (40.3)	18/55 (32.7)	(-0.18, -0.28 − -0.07)	(0.08, -0.07 − 0.20)	(0.08, -0.07 − 0.20)

*Note*: True positive cases who submitted a urine sample and met the case definition according to WHO 2009 guidelines (n = 513)

*Wilson score intervals for differences of proportions between Dengue and Dengue with warning signs and 95% confidence interval (CI)

Ɨ Wilson score intervals for differences of proportions between Dengue and Severe Dengue and 95% CI

ǂ Wilson score intervals for differences of proportions between Dengue with warning signs and Severe Dengue and 95% CI

## Discussion

The primary objective of this study was to evaluate the utility of urine anti-DENV IgM in diagnosing dengue cases among patients with AFIs. The advantage of using urine as a surrogate diagnostic specimen is the relative ease and low cost associated with sample collection as well as high patient acceptability. We chose anti-DENV IgM as the analyte because antibody tests are relatively inexpensive, and they are rapid and simple to perform in the acute care setting. Unfortunately, our study found that urine anti-DENV IgM ELISA was not a sensitive method to diagnose DENV infections when compared with serum assays among patients of with an AFI, but it was highly specific. Previous studies that evaluated anti-DENV IgM have been able to detect no anti-DENV IgM in the urine of dengue patients [4, 5]. These studies differed from our study in that they used different immunoassays including a commercial IgM assay from Bio-Rad [5] and an in-house assay [4]. In addition, their dengue cases were primarily mild, dengue fever cases whereas our study population had more severe disease with most cases meeting criteria for either severe dengue or dengue with warning signs.

A secondary objective was to determine if urine anti-DENV IgM positivity varied by dengue severity. Our study found that urine anti-DENV IgM positivity was more common among hospitalized participants and those with abnormal clinical laboratory results consistent with severe dengue. Our study suggests that urine anti-DENV IgM may be marker for severity early in the clinical course. However, more evaluation is needed since our sample size was relatively modest, and we found that more than half of all participants regardless of severity had anti-DENV IgM detectable late in the clinical course. Interestingly, a recent study by Zhao *et al*., found that the presence of anti-DENV IgA in urine was associated with severe dengue, with 68.4% (13/19) severe dengue cases versus 32.6% (15/46) non-severe cases having IgA detected in the urine 4 to 7 DPO [8].

Compared to IgA, IgM is a large pentamer (molecular radius 120 A°) that is only able to pass the glomerular filtration barrier through large defects and shunts, and therefore detecting IgM in urine may indicate dysfunction of the glomerular filtration barrier [12]. Recent studies have suggested that the mechanism of vascular leakage observed in severe dengue cases is due to the disruption of the endothelial glycocalyx layer (EGL) by dengue non-structural protein 1 (NS1) [28, 29]. In fact, a clinical study by Suwarto *et al*., found that two of the four components of EGL were elevated in the serum of severe dengue patients [30]. In addition, studies have demonstrated evidence of glomerular changes in patients with dengue hemorrhagic fever [31], and medical complications including AKI, glomerulonephritis and acute renal failure have been described among dengue patients [15]. While chronic conditions such as diabetes and sickle cell disease have associated nephropathies that involved urinary IgM excretion [10, 32, 33], our study did not find an association between chronic diseases and urine anti-DENV IgM positivity.

This study had several limitations. We must be cautious in our interpretation of the presence of anti-DENV IgM in urine as an indicator for severe disease because this study had a relatively small sample size for dengue (n = 73) and severe dengue (n = 55) compared to dengue with warning signs (n = 385). Another limitation was that the study only collected specimens for 10 months corresponding to only one dengue transmission season of which most cases were identified as DENV-1. Finally, to validate urine as a sample type, we performed a spike and recovery test not a full validation study.

In conclusion, the use of urine as a surrogate specimen for dengue diagnosis was not sensitive in comparison to serum; however, the presence of anti-DENV IgM urine was highly specific. We were able to identify the presence of anti-DENV IgM in urine specimens from laboratory-positive dengue cases using a simple diagnostic assay. This assay may prove to be useful as a diagnostic tool as well as a prognostic tool to differentiate dengue from dengue with warning signs early in the course of illness (DPO<5). However, given what we now know about NS1 and endothelial glycocalyx disruption, future studies should include NS1 testing and use a larger sample size of dengue and severe dengue early in the course of illness are needed to better understand the utility of this assay to predict disease severity.

### Disclaimers

The opinions expressed by authors contributing to this journal article do not necessarily reflect the opinions of the Centers for Disease Control and Prevention or the institutions with which the authors are affiliated.

## Supporting information

S1 Table Comparison of the presence of anti-DENV IgM in urine between males and females for each age group. (DOCX)

S2 Table Checklist STROBE statement. (DOC)
