# Supplementary material for: The detection of anti-dengue virus IgM in urine in participants enrolled in an acute febrile illness study in Puerto Rico
Source: PLoS Negl Trop Dis. 2020 Jan 29;14(1):e0007971. doi: 10.1371/journal.pntd.0007971 (PMC6988914; doi:10.1371/journal.pntd.0007971)
Supplement: S1 Table — (DOCX) [file pntd.0007971.s001.docx]

**S1 Table. Comparison of the presence of anti-DENV IgM in urine between males and females for each age group**

a Age < 9 years

b Age 45+ years

* Pearson Chi-Square test to compare proportions
